# One-year daily consumption of buttermilk drink containing lutein-enriched egg-yolks does not affect endothelial function in fasting and postprandial state

**DOI:** 10.1038/s41598-017-01370-7

**Published:** 2017-05-02

**Authors:** Sanne M. van der Made, Tos T. J. M. Berendschot, Aize Kijlstra, Jogchum Plat

**Affiliations:** 1 Department of Human Biology and Movement Sciences, NUTRIM School for Nutrition, Toxicology and Metabolism, Maastricht University Medical Centre, 6200 MD Maastricht, The Netherlands; 2 University Eye Clinic Maastricht, Maastricht University Medical Centre, 6202 AZ Maastricht, Maastricht The Netherlands

## Abstract

Previous results have shown that one-year daily consumption of a lutein-enriched egg yolk containing dairy drink did not significantly affect fasting serum lipid and lipoprotein concentrations in adults with early signs of macular degeneration. The current study further substantiates these findings with parameters reflecting endothelial function. Additionally, we extend our observations from the fasting to the postprandial situation. Subjects participated in a 1-y randomized placebo-controlled dietary intervention trial. 52 subjects were included in the active (Egg) group and 49 in the control (Con) group. Changes in postprandial biochemistry (triacylglycerol (TAG), glucose and non-esterified fatty acids (NEFA)) following a mixed meal and flow-mediated dilation (FMD) analyses were evaluated at the start and after one year intervention. Postprandial glycemic and lipemic responses before the intervention as well as the differences in postprandial responses after one-year intervention were comparable between the Egg and the Con group. Fasting FMD was comparable between the groups before the intervention started and at the end of intervention. Additionally, the change in FMD following a mixed meal was comparable between the groups. To conclude, one-year consumption of a lutein-enriched egg yolk incorporated in a dairy drink has no effect on postprandial lipid and glucose metabolism or endothelial function.

## Introduction

Eggs are an inexpensive, rich source of high-quality protein, essential fatty acids, minerals, and vitamins A, B12, D, K and folate. Additionally, eggs contain relatively high amounts of cholesterol and lutein. Lutein is a xanthophyll that is concentrated in the human retina where it plays an important role in maintaining visual function. It is not synthesized in the human body and intake depends on dietary sources such as eggs and green leafy vegetables.

Elevated intakes of other dietary components, such as cholesterol and saturated fatty acids (SAFAs), are known to increase serum LDL cholesterol concentrations and as such may impair endothelial function^1^. In view of the fact that an elevated intake of dietary cholesterol has been shown to increase serum LDL cholesterol^2^ and the total:HDL cholesterol ratio^3^, the intake of egg(yolk)s has been discouraged over the past decades and dietary guidelines advice to limit SAFA intake to a maximum of 10en%^4^. However, evidence - mainly from large epidemiological (prospective cohort) studies - has not shown a significant relationship between egg intake and CVD^5–7^. Egg yolks are not only rich in cholesterol but also contain lutein, a xanthophyll carotenoid with antioxidant and anti-inflammatory properties. Serum carotenoid concentrations have been inversely associated with biomarkers for cardiovascular disease risk prediction^8, 9^ and serum asymmetric dimethylarginine (ADMA) concentrations^10^. Moreover, a Chinese study recently showed that a one-year supplementation of lutein improved carotid IMT in subjects with subclinical atherosclerosis^11^.

The intervention used in this study is a combination between traditionally prepared buttermilk, which is the liquid left over after churning butter from cream, and lutein-enriched egg yolks. It was hypothesized that traditionally prepared buttermilk prevents the anticipated increase in serum cholesterol concentrations when consuming 1.5 egg yolks daily. This hypothesis has been tested and was reported in a separate study^12^.

Whether the intake of a lutein-enriched egg drink affects endothelial function, considering both an increased lutein and dietary cholesterol intake, has not yet been investigated and was the purpose of the study described here.

Recently, we showed that one-year daily consumption of a buttermilk drink containing lutein-enriched egg-yolk did not significantly change fasting serum lipid or (apo)lipoprotein concentrations, whereas plasma lutein concentrations were markedly increased^13^. However, the fact that serum cholesterol concentrations were not significantly elevated does not exclude the possibility that endothelial function might have been affected. Evidence for this statement comes from earlier studies in mice that reported a decreased endothelial function in animals fed a high-cholesterol diet despite the fact that circulating serum cholesterol concentrations were hardly affected^14^. Therefore, although fasting lipid profiles are of great value in CVD risk predictions, we here further evaluated the effects on a broader panel of cardiovascular risk parameters in the same study population before and after one-year daily consumption of lutein-enriched egg-yolks incorporated in a dairy drink. Moreover, we measured the capability of the body to adapt to a high fat mixed meal challenge by evaluating changes in postprandial lipid and glucose metabolism and endothelial function, measured by flow-mediated dilation.

## Subjects and Methods

### Subjects

One hundred and one (N = 101) subjects were recruited for a one-year double blind placebo-controlled intervention trial with a parallel design, which was approved by the Medical Ethics Committee of Maastricht University Medical Centre and registered at clinicaltrials.gov as NCT00902408 on 19 May 2009. Screening for eligibility started in July 2009, final measurements were performed in December 2011. Subjects were recruited via advertisements in newspapers in the province of Limburg, The Netherlands. The trial adhered to the tenets of the declaration of Helsinki and before the screening procedure started, written informed consent was obtained from all subjects. The main inclusion criteria were being at least 50 years of age, not receiving treatment for diabetes or elevated serum cholesterol concentrations and having early signs of macular degeneration, as evidenced by drusen and/or retinal pigment epithelium alterations in at least one eye assessed through fundus photography. Next to this, it was not allowed to use dietary supplements containing lutein or zeaxanthin. Subjects allergic to eggs or egg-products were not included.

During the 1-year follow-up of the study, 12 participants withdrew (six from the experimental group (Egg) and six from the control (Con) group). One subject that completed the study was omitted from further statistical evaluations because of outlying HDL cholesterol concentrations, as evidenced by a large Cook’s distance. The flow of participants through the study and reasons for withdrawal (which were not related to the study product as such) can be found in Supplemental Figure 1.

### Study design and products

The study had a randomized, double blind, placebo-controlled parallel design with a duration of one year. Subjects were randomly assigned to either the Con or Egg group before starting the study according to a pre-established, computer generated randomization scheme. Allocation was concealed in sequentially numbered, sealed envelopes and stored by the study coordinator. The intervention consisted of a lutein-enriched egg-yolk based dairy drink (provided by Newtricious R&D, Oirlo, The Netherlands). Egg-yolks were enriched through natural sources, via the feed of laying hens. This feed complied with the EU regulation concerning the maximal xanthophyll level of 80 ppm. One and a half enriched egg-yolks were then incorporated into an 80 mL buttermilk drink that provided on average 1.4 mg lutein and 323 mg cholesterol per day. Subjects allocated to the Con group received a comparable buttermilk drink, without the addition of egg yolks. The color of the control drink was matched to the lutein-enriched egg drink through addition of synthetic colorants E104 and E110. The nutrient composition of both drinks can be found in Supplemental Table 1.

Subjects participated in a mixed meal challenge at the start of the study and again after one-year consumption of either the control or lutein-enriched egg-drinks. The high-fat mixed meal consisted of two muffins, with a total macronutrient composition as presented in Supplemental Table 2. One batch of muffins was prepared for the entire study and stored at −20 °C until consumption. On the postprandial test day, subjects arrived at university after an overnight fast. First an FMD measurement was performed, followed by insertion of an intravenous cannula (Becton, Dickinson and Company, Franklin Lanes, NY, USA) in an antecubital vein and collection of a fasting (T0) blood sample. Hereafter, subjects were asked to consume the mixed meal within 10 min. Subsequent blood samples were drawn 15 (T15), 30 (T30), 45 (T45), 60 (T60), 90 (T90), 120 (T120), and 240 (T240) min after mixed meal consumption. Subjects were allowed to drink water at their convenience throughout the entire postprandial test. Additionally, subjects were asked to fill out a validated food frequency questionnaire at baseline and at the end of the intervention period, as reported elsewhere^13^.

### Flow-mediated dilation

Before and 4 h after consuming the mixed meal, FMD measurements were performed according to standard guidelines^15^ to assess endothelium-dependent vascular reactivity. The same ultrasonographer performed all measurements by using a high-resolution ultrasound system with a 3–11 MHz wide band linear array transducer (Philips Sonos 5500 System; Philips Ultrasound, Andover, MA, USA). After at least 15-min supine rest in a quiet, temperature-controlled room, images of the brachial artery were taken longitudinally, 2–10 cm above the antecubital fossa in combined Doppler/brightness mode (B-mode). A probe holder was used to secure the position of the transducer during the measurement. After a 3 min baseline measurement, a peripherally hypoxic state was induced by inflation of a pneumatic tourniquet (Hokanson TD312 automatic cuff inflator, D.E. Hokanson, Inc. WA, USA) placed around the forearm, inflated to a pressure of at least 50 mmHg above systolic pressure, with a minimal pressure of 200 mmHg. After 5 minutes, the cuff was deflated, resulting in reactive hyperemia, which was recorded for another 5 minutes. Images were recorded on DVD continuously during the entire 13 min measurement protocol. Acquired images were analyzed using an in-house developed semi-automated image-analysis algorithm (Dept. of Biomedical Engineering, Maastricht University Medical Center, The Netherlands). FMD response was expressed as the percentage increase in brachial artery diameter from baseline to maximal dilation post-occlusion.

### Blood sampling and analyses

Blood was sampled into NaF-containing tubes and serum separator tubes (Becton, Dickinson and Company, Franklin Lanes, NY, USA) at the indicated time points, which were in between the two FMD measurements at baseline and after 240 minutes. NaF-containing tubes were immediately placed on ice after blood sampling, followed by centrifugation at 1300 × *g* for 15 min at 4 °C. Serum separator tubes were allowed to clot for at least 30 min at 21 °C before centrifugation at 1300 × *g* for 15 min at 21 °C. Plasma and serum aliquots were directly snap-frozen in liquid nitrogen and stored at −80 °C until analysis.

Plasma glucose concentrations were measured in NaF plasma at T0, 15, 30, 45, 60, 90, 120 and 240 (Horiba ABX, Montpellier, France). Non-esterified fatty acid (NEFA) concentrations were also analyzed in NaF plasma (NEFA kit; WAKO Chemicals, Germany) at the indicated time points. Concentrations of TAG, corrected for free glycerol, were determined in serum samples from T0, 15, 30, 45, 60, 120 and 240 (GPO Trinder; Sigma Diagnostics).

Fasting serum samples were analyzed for total cholesterol, HDL cholesterol, TAG and apolipoprotein A-I (apoA1) and apolipoprotein B100 (apoB100) concentrations. LDL cholesterol concentrations were calculated according to the Friedewald equation^16^.

### Statistical analyses

All results are presented as mean ± SD, unless otherwise indicated. Differences in baseline concentrations as well as changes in metabolic risk parameters and FMD between the Egg and Con group were tested using an independent samples student’s t-test. Pearson correlation coefficients were used to calculate correlations. Postprandial glucose, TAG and NEFA changes were assessed by calculating differences within a subject between baseline and the end of intervention for each postprandial time point. Differences between Egg and Con group were assessed by linear mixed model (LMM) analyses with treatment and time point as within-subject fixed factors and treatment × time as interaction term. If the interaction term was not significant, it was omitted from the model. If factor time was significant, post hoc tests with Bonferroni correction were carried out to compare concentrations to baseline concentrations. Change between FMD responses before and after a mixed meal at baseline were substracted from the response before and after a mixed meal after the one-year intervention. Differences between these responses were evaluated by an independent samples student’s T-test.

As the primary outcome of this study was macular pigment optical density (MPOD), statistical power calculation was initially based on this parameter^17^.

Differences were considered statistically significant if P < 0.05. All statistical analyses were performed using SPSS 21.0 for Mac Os X (SPSS Inc., Chicago, IL, USA).

## Results

### Study participants and dietary intake

The flow of participants throughout the study including reasons for drop out is shown in Supplemental Figure 1. Baseline characteristics of the eighty-nine subjects that completed the study are shown in Table 1. One subject was omitted from final analyses because of outlying HDL cholesterol concentrations. Therefore, results in metabolic parameters are shown for eighty-eight subjects (N = 45 in the Egg and N = 43 in the Con group). Within this group of eighty-eight subjects, there were technical problems during the FMD measurement on one of the test-days, which hampered proper analysis in five subjects (one in the Con group and four in the Egg group). In addition, eight subjects (four in both groups) were omitted from the statistical analysis on FMD, because of poor quality of the FMD measurements. Therefore, FMD results are shown for seventy-five subjects (N = 40 in the Egg and N = 35 in the Con group).

**Table 1:** Baseline characteristics, serum lipids and (apo)lipoproteins, markers for cholesterol metabolism and liver- and kidney function of study participants within the egg-drink (Egg) and control (Con) groups^1^.

	Egg (N = 45)	Con (N = 43)
Sex (M/F)	15/30	14/29
Age (y)	62 ± 6	62 ± 8
Smoking (yes/no)^2^	18/26	16/27
BMI (kg/m^2^)	26.9 ± 3.6	26.3 ± 3.7
Systolic blood pressure (mmHg)	133 ± 18	136 ± 23
Diastolic blood pressure (mmHg)	76 ± 9	78 ± 11
Flow-mediated dilation (%)^3^	2.48 ± 1.74	2.59 ± 2.20
Serum total cholesterol (mmol/L)	6.01 ± 1.14	6.04 ± 1.15
Serum LDL cholesterol (mmol/L)	3.82 ± 1.06	3.87 ± 0.98
Serum HDL cholesterol (mmol/L)	1.65 ± 0.41	1.67 ± 0.39
Serum apoB100 (g/L)	0.99 ± 0.24	0.99 ± 0.22
Serum apoAI (g/L)	1.38 ± 0.21	1.38 ± 0.21
Serum TAG (mmol/L)	1.18 ± 0.63	1.09 ± 0.45
Plasma glucose (mmol/L)	5.46 ± 0.43	5.41 ± 0.46
Serum hsCRP (mg/L)	2.49 ± 4.13	1.96 ± 2.17
Plasma NEFA (μmol/L)	503 ± 167	518 ± 209
Serum creatinine (μmol/L)	70.8 ± 13.4	75.0 ± 12.4
Serum ALP (IU/L)	74.9 ± 16.6	84.3 ± 22.6*
Serum GGT (IU/L)	28.2 ± 16.7	24.7 ± 12.6
Serum ASAT (IU/L)	19.6 ± 7.2	22.6 ± 12.4
Serum ALAT (IU/L)	21.6 ± 6.6	23.9 ± 12.6
Serum bilirubin (μmol/L)	15.0 ± 4.5	16.6 ± 7.1

^1^Values are means ± SDs. *Different from Con, P < 0.05. ALAT; Alanine transaminase, ALP; Alkaline phosphatase, ASAT; Aspartate transaminase, CRP; high-sensitivity C-reactive protein, GGT; Gamma-glutamyltranspeptidase, NEFA; non-esterified fatty acids. ^2^Smoking status of one subject in the Egg group was not provided. ^3^Flow-mediated dilation results are shown for N = 42 in the Egg group and N = 39 in the Con group.

At baseline energy intake and the proportion of energy from carbohydrates, total fat and protein as well as intake of micronutrients was comparable between the Egg and Con group. Apart from a higher dietary cholesterol intake in the Egg group compared to the Con group (P < 0.001) and a lower oleic acid intake in the Egg group compared to the control group (P < 0.05) during the intervention, no differences were observed in total energy and nutrient intake during the study^13^.

### Fasting lipids, (apo)lipoproteins and FMD

**Figure 1:**
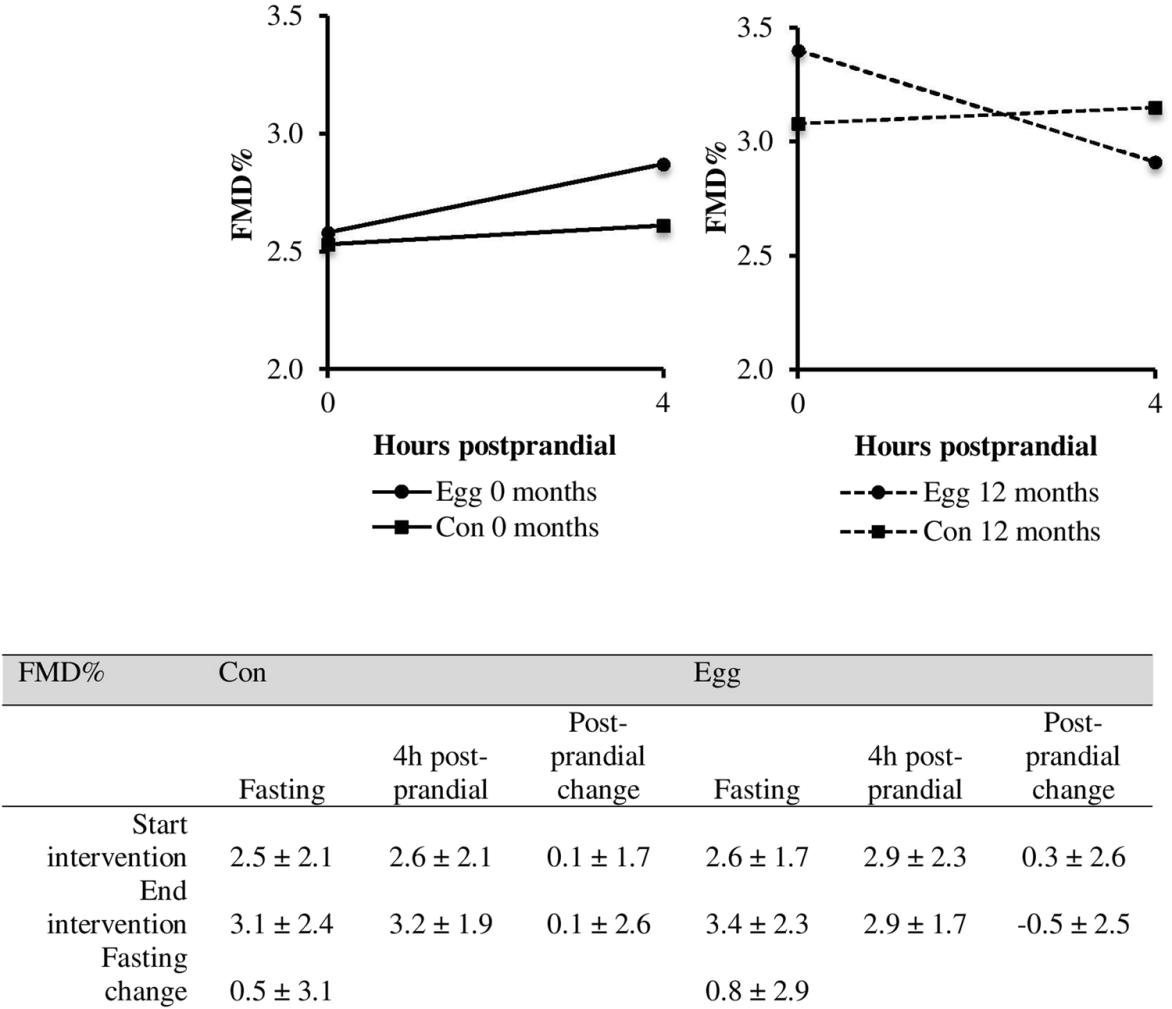
Change in FMD% measured at the start of intervention (0 months, solid lines) and at the end of intervention (12 months, dashed lines) in the Egg group (rounds) and in the Con group (squares). No significant differences were found. Exact FMD values are given in the accompanying table.

As described earlier, no significant changes were found in fasting serum total-, HDL- and LDL cholesterol, total-to-HDL cholesterol ratio, apoB100, apoAI, TAG and NEFAs between the Egg and Con group over the 1-year intervention^13^. As shown in Fig. 1, no significant difference was found in fasting FMD between the groups at the start of intervention (Egg vs. Con; 2.6 ± 1.7% vs. 2.5 ± 2.1%, P = 0.92). Also, no significant change was observed in blood pressure or BMI. Moreover, there were no significant correlations between any of the metabolic parameters and FMD (data not shown).

### Postprandial glycaemia and lipemia

**Figure 2:**
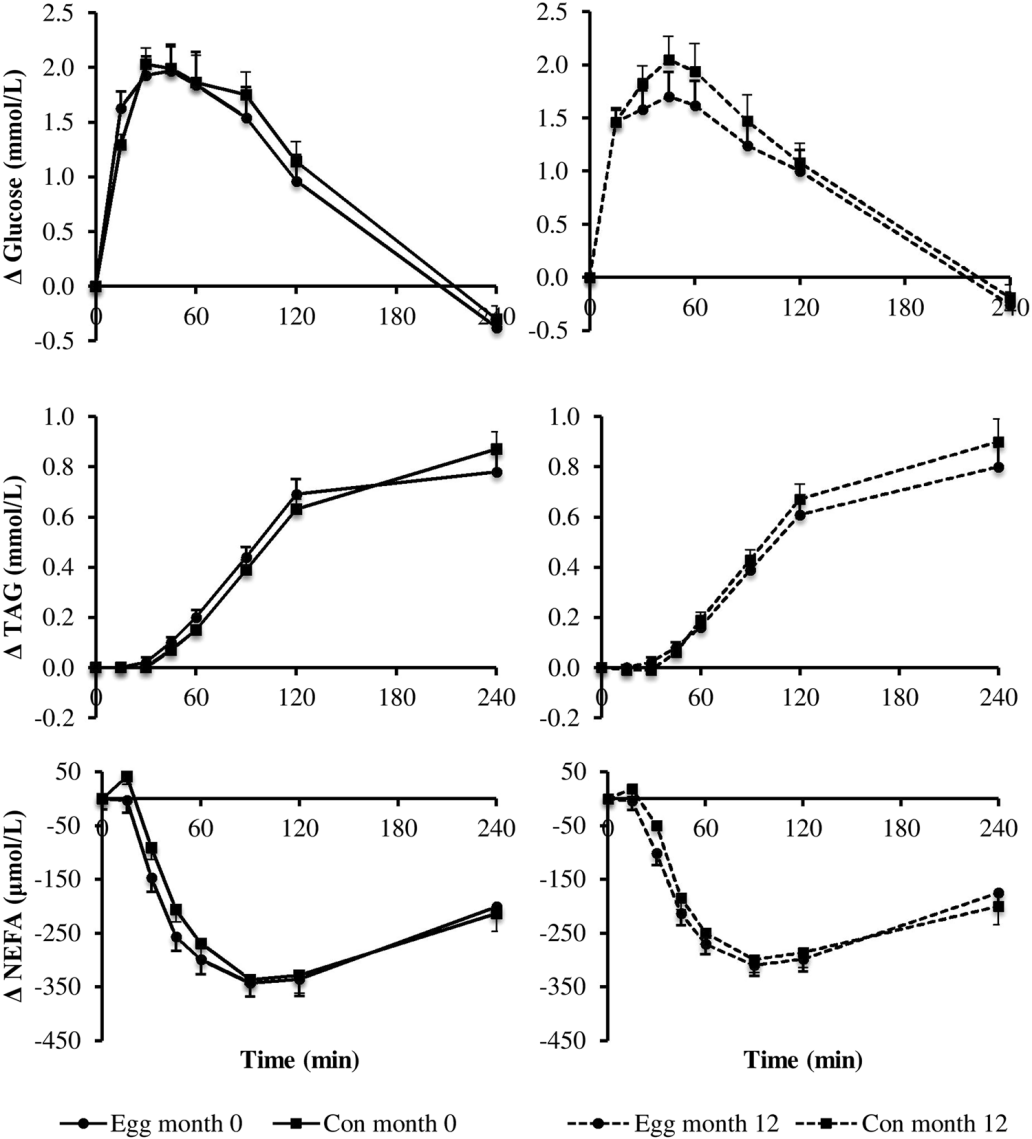
Mean changes (±SEM) in plasma glucose, serum triacylglycerol (TAG) and non-esterified fatty acid (NEFA) concentrations following a mixed meal at the start of intervention (month 0) and at the end of intervention (month 12) in a parallel study in subjects with early signs of macular degeneration. Analyses were performed in 45 subjects in the Egg group and 43 subjects in the Con group. Differences between Egg and Con groups were analyzed using Linear Mixed Models. There were no significant meal × time interactions for NEFA, TAG and glucose. Time effects were P < 0.05 for NEFA and glucose. No effects of intervention were found.

Fasting concentrations of NEFA, glucose and TAG were not different between the Con and the Egg group (Fig. 2). Glucose and TAG concentrations increased and NEFA decreased significantly after mixed meal consumption, both before and after intervention (P < 0.001 for time effect). The change in postprandial glucose, NEFA and TAG was not different between the Egg and Con group after one year of consuming the lutein-enriched egg drink. However, a significant difference between the Egg and Con group regarding the factor time was found for glucose and NEFA (P = 0.027, P = 0.002). This implies that these postprandial responses are different between both groups, irrespective of the intervention. Post-hoc comparisons did not reveal which time point was exactly different from baseline in either the glucose or the NEFA response.

### Postprandial FMD

At the start of intervention, the postprandial change in FMD was not significantly different between the groups; i.e a worsening of 0.1 ± 1.7% in the Con and of 0.3 ± 2.6% in the Egg group. After 12 months intervention, fasting FMD increased by 0.5 ± 3.1% in the Con group and 0.8 ± 2.9% in the Egg group as compared to the start of the intervention, however these changes were not significantly different between the groups. Also, the change in FMD after the mixed meal was not different between the Egg (−0.5 ± 2.5%) and Con (0.1 ± 2.6%) groups. Most importantly, the change in FMD after a mixed meal before and after one-year intake of lutein-enriched egg-drinks was not different from the controls receiving a placebo dairy drink (Egg vs. Con; −0.8 ± 3.3 vs. 0.0 ± 3.5%, P = 0.33).

## Discussion

This study shows that during a period of one year, daily consumption of a dairy drink containing 1.5 lutein-enriched egg-yolks, does not affect postprandial glycemic and lipemic responses and endothelial function as measured by flow-mediated dilation.

Over the past decades, the effect of dietary cholesterol in general and egg consumption in particular on cardiovascular outcomes has been a controversial issue. The reason for potential concerns is the well-defined serum LDL cholesterol raising effect of increased dietary cholesterol intake^18^. As shown earlier, an increase in LDL cholesterol concentration of 0.16 ± 0.01 mmol/L was expected after 1-y consumption of the lutein-enriched egg-drink^13^. In a healthy population, with average serum LDL cholesterol concentrations of 3 mmol/l, this means an increase in serum LDL cholesterol of approximately 5%. Based on estimations as described in a meta-analysis from the Cholesterol Treatment Trialists’ Collaboration, this would translate in an increased risk for major vascular events of ±5%^18^. However, epidemiologic studies evaluating the effect of egg consumption on (intermediary) outcomes of CVD are not conclusive^5–7, 19, 20^. Moreover, the long-term effect of increased dietary cholesterol intake on intermediate cardiovascular endpoints has not been assessed in a randomized clinical trial. Endothelial dysfunction, as assessed by FMD in the present study, is an early hallmark in the process of atherosclerotic plaque development^21^, which therefore might act as intermediate cardiovascular endpoint. Earlier studies did not observe an effect of short-term daily intake of eggs on endothelial function and lipid profile in both healthy^22^ and hyperlipidemic^23^ subjects. Our results extend these findings and show that also long-term consumption of lutein-enriched egg yolks incorporated in a buttermilk drink had no effect on endothelial function as measured by FMD in a population of slightly hypercholesterolemic, overweight male and female subjects with early signs of macular degeneration.

It should be mentioned that the egg yolks used in this study were enriched in lutein, a carotenoid that is hypothesized to be protective against early atherosclerosis^24^. An *in vitro* study revealed that lutein was able to improve endothelial function through increasing NO and decreasing the release of endothelin^25^. Furthermore, lutein downregulated NF-κB signaling, and decreased several markers for endothelial activation and inflammation. Also, a significant inverse relation was observed between plasma soluble intercellular adhesion molecule-1 (sICAM-1) and lutein concentrations in a cross-sectional study in a general population sample^26^. Recently, a study from China showed that one-year daily supplementation of 20 mg lutein improved carotid IMT in subjects with subclinical atherosclerosis^11^. Therefore, it might be possible that the combined intake of dietary cholesterol with an increased quantity of lutein counterbalanced each other’s effects on flow-mediated dilation. This also implies that the observation that one year daily intake of these lutein-enriched egg-yolks did not affect endothelial function cannot be extrapolated to regular eggs of which the yolks are not enriched in lutein. In view of the difficulties in obtaining a large sample size of individuals with early signs of age-related macular degeneration we decided not to include a reference group that consumed regular eggs for one year in our trial. Moreover, the primary goal of our trial was to find out whether intake of lutein-enriched egg drinks were able to increase circulating lutein levels and visual function, which was addressed in a separate paper^17^.

Another explanation for not finding an effect on endothelial function, after consuming one-and-a half egg yolks incorporated in a buttermilk drink that provided on average 323 mg dietary cholesterol additionally per day for one year, might be caused by the fact that egg consumption is thought to mainly increase particularly the larger, more buoyant, LDL (LDL-1) particles. These particles are thought to be less atherogenic than small (LDL-3+) particles, because, compared to smaller LDL particles, they are not able to enter the arterial wall as easily, their binding capacity to proteoglycans is not enhanced, they are less susceptible to oxidation and their affinity to bind to the LDL receptor is not decreased^27^. These features might explain the fact that small LDL particles are related to endothelial dysfunction in several groups of subjects, which is not the case for larger LDL particles^28–30^. However, LDL subclass distribution was not measured in our subjects. In spite of the postprandial rise in glucose, TG and NEFA concentrations, no significant deterioration in 4h-postprandial FMD was observed in the current study. This observation is not in line with research that shows a decrease in FMD after consuming a comparable high-fat mixed meal^31^. This might be due to the moment of FMD measurement, as this study shows a pronounced deterioration 2 h postprandially, whereas FMD was measured 4 hours postprandially in the current study.

In conclusion, we here show that daily consumption of a dairy drink containing lutein-enriched egg-yolks had no adverse effect on endothelial function or postprandial glucose and lipid metabolism in subjects with early signs of macular degeneration.

## Electronic supplementary material

Supplementary info
