# Supplementary material for: One-year daily consumption of buttermilk drink containing lutein-enriched egg-yolks does not affect endothelial function in fasting and postprandial state
Source: Sci Rep. 2017 May 2;7:1353. doi: 10.1038/s41598-017-01370-7 (PMC5431059; doi:10.1038/s41598-017-01370-7)
Supplement: Supplementary file 1 — Supplementary info [file 41598_2017_1370_MOESM1_ESM.doc]

One-year daily consumption of buttermilk drink containing lutein-enriched egg-yolks does not affect endothelial function in fasting and postprandial state

Sanne M. van der Made, Tos T.J.M. Berendschot, Aize Kijlstra, and Jogchum Plat

**Enrollment**

Assessed for eligibility (n=284)

Excluded (n=184)

- Did not meeting inclusion criteria (n=184)

Randomized (n=101)

**Allocation**

**Analysis**

Allocated to Egg group (n=52)

Allocated to Con group (n=49)

**Follow-Up**

Discontinued intervention (n=6), reasons:

- Started taking supplements (n=1)
- Dislike of study product (n=1)
- Stomach complaints (n=1)
- Breast complaints (n=2)
- Intestinal complaints (n=1)

Discontinued intervention (n=6), reasons:

- Dislike of study product (n=1)
- Started statin treatment (n=2)
- Globus nervosus (n=1)
- Stomach complaints (n=1)
- Family circumstances (n=1)

Analysed (n=43)

Analysed (n=45)

Excluded from analysis because of:

- outlying HDL cholesterol concentrations (n=1)

**SUPPLEMENTAL FIGURE 1** Subject flow chart

| **SUPPLEMENTAL TABLE 2** Nutrient composition of muffins (serving of 2) | |  |
| --- | --- | --- |
|  | **Muffins** | |
| Energy (kJ) | 4095 | |
| Energy (kcal) | 980 | |
| Protein (g) | 14.5 | |
| Carbohydrates (g) | 103.0 | |
| Total fat (g) | 56.6 | |
| SFA (g) | 33.9 | |
| TransFA (g) | 2.2 | |
| MUFA (g) | 14.5 | |
| PUFA (g) | 2.7 | |
| Cholesterol (mg) | 349 | |

| **SUPPLEMENTAL TABLE 1** Nutrient composition of control- and lutein-enriched egg-yolk drink (per daily 80 mL dosage) | | |
| --- | --- | --- |
|  | **Control drink** | **Lutein-enriched egg-yolk drink** |
| Energy (kcal) | 41 | 131 |
| Fat (g) | 0.4 | 9.2 |
| SFA (%) | 67.0 | 32.5 |
| TransFA (%) | 3.1 | 0.3 |
| MUFA (%) | 26.2 | 49.5 |
| PUFA (%) | 3.7 | 17.7 |
| Protein (g) | 2.7 | 6.2 |
| Carbohydrate (g) | 6.6 | 5.8 |
| Cholesterol (mg) | 2.0 | 323 |
| Lutein (mg) | - | 1.4 |
| Zeaxanthin (mg) | - | 0.3 |
